# Supplementary material for: Reconciling Mining with the Conservation of Cave Biodiversity: A Quantitative Baseline to Help Establish Conservation Priorities
Source: PLoS One. 2016 Dec 20;11(12):e0168348. doi: 10.1371/journal.pone.0168348 (PMC5173368; doi:10.1371/journal.pone.0168348)
Supplement: S1 Dataset — (ZIP) [file pone.0168348.s002.zip › Taxa/Serra Norte/SN_2013/N4WS/Lista N4WS-80.pdf]

CAVIDADE N4WS-0080

Classe	Ordem	Fam/Outros	Gên/Outros	Espécie	S-U	U-S	Zon.
Arachnida	Acari	(Parasitengonina)		sp.6	X	X	P
Arachnida	Acari	Ixodidae	*Amblyomma*	sp.1		X	P
Arachnida	Araneae	Filistatidae		jovem	X		P
Arachnida	Araneae	Ochyroceratidae	*Ochyrocera*	sp.2	X	X	P
Arachnida	Araneae	Pholcidae		jovem	X		E
Arachnida	Araneae	Scytodidae	*Scytodes*	jovem	1		P
Arachnida	Araneae	Segestriidae	*Ariadna*	sp.1		X	P
Arachnida	Araneae	Theridiidae	*Achaearanea*	sp.1	X	X	E/P
Arachnida	Araneae	Theridiosomatidae	*Plato*	sp.1	X	X	E/P
Chilopoda	Scolopendromorpha			sp.	1		P
Chilopoda	Scutigeromorpha	Pselliodidae		jovem		1	P
Diplopoda	Spirostreptida	Pseudonannolenidae	*Pseudonannolene*	sp.1		2	P
Entognatha	Collembola	Entomobryidae		sp.6		X	P
Entognatha	Collembola	Paronellidae		sp.1	X		E
Entognatha	Diplura	Campodeidae		sp.1	X	X	E/P
Insecta	Blattodea			jovem		1	P
Insecta	Coleoptera	Staphylinidae	Pselaphinae	sp.14		X	P
Insecta	Coleoptera	Staphylinidae	Pselaphinae	sp.16	X		E
Insecta	Coleoptera	Staphylinidae	Pselaphinae	sp.20		X	P
Insecta	Diptera	Cecidomyiidae	Cecidomyiinae	sp.	X		E
Insecta	Diptera	Chironomidae		sp.	X		E
Insecta	Diptera	Dolichopodidae		sp.	X		E
Insecta	Diptera	Psychodidae	*Sciopemyia*	*sordellii*	X	X	P
Insecta	Heteroptera	Reduviidae	Emesinae	jovem	2		P
Insecta	Homoptera	Cercopidae		jovem		X	E
Insecta	Homoptera	Cixiidae		sp.1	X	X	P
Insecta	Homoptera	Cixiidae		sp.3	X		E
Insecta	Hymenoptera	Formicidae	*Camponotus*	sp.1		X	P
Insecta	Hymenoptera	Formicidae	*Gnamptogenys*	*striatula*	X		E
Insecta	Hymenoptera	Formicidae	*Hypoponera*	sp.1	X	X	P
Insecta	Isoptera			sp.	X	X	P
Insecta	Lepidoptera	Gelechioidea		sp.1	X		E/P
Insecta	Lepidoptera	Glossata		sp.2		X	E
Insecta	Lepidoptera	Noctuoidea		sp.1		8	E
Insecta	Lepidoptera	Tineoidea		sp.4		X	E
Insecta	Neuroptera	Myrmeleontidae		jovem	2	2	E
Insecta	Orthoptera	Phalangopsidae	*Paraclodes*	sp.	1		E
Turbellaria	Tricladida			sp.2	X		P
Amphibia	Anura	Strabomantidae	*Pristimantis*	cf. *fenestratus*	3	5	E/P
